# Genetic Interactions Between Arabidopsis *DET1* and *UVH6* During Development and Abiotic Stress Response

**DOI:** 10.1534/g3.112.003368

**Published:** 2012-08-01

**Authors:** Esther Kim, Valentina Ly, Avril Hatherell, Dana F. Schroeder

**Affiliations:** Department of Biological Sciences, University of Manitoba, Winnipeg, MB R3T 2N2, Canada

**Keywords:** Arabidopsis, *DET1*, *UVH6*, light, heat

## Abstract

Plants must adapt to a variety of abiotic inputs, including visible light, ultraviolet (UV) light, and heat. In *Arabidopsis thaliana*, *DE-ETIOLATED 1 (DET1)* plays a role in visible light signaling, UV tolerance, and development. *UV-HYPERSENSITIVE 6* (*UVH6)* mutants are UV and heat sensitive, as well as dwarf and pale, like *det1*. In this study, we examine the genetic interactions between these two genes. In dark-grown seedlings, *uvh6* exhibits a weak de-etiolated phenotype but does not affect the stronger de-etiolated phenotype of *det1*. In the light, *det1* is epistatic to *uvh6* with regard to chlorophyll level, but their effect on all size parameters is additive and therefore independent. With regard to UV tolerance, *det1* UV resistance is epistatic to *uvh6* UV sensitivity. In heat stress experiments, *det1* enhances heat-induced tissue damage in the *uvh6* background but suppresses heat-induced growth inhibition. Thus, *det1* acts epistatically to *uvh6* with respect to de-etiolation, chlorophyll level, UV tolerance, and heat-induced growth inhibition, whereas *det1* and *uvh6* act additively to regulate plant size and heat-induced cell death. These data provide insight into interplay between light and heat signaling.

Plants are unable to move and thus must respond appropriately to their environment. The sun is a key component of a plant's environment, providing visible light for photosynthesis but also generating damaging ultraviolet (UV) rays and heat. In this study, we examine the role of two genes, *DE-ETIOLATED 1 (DET1)* and *UV-HYPERSENSITIVE 6* (*UVH6)*, in plant response to visible light, UV, and heat.

Light provides essential information during plant development. A dramatic example of the effect of light can be seen during seedling growth. Seedlings grown in the light have short hypocotyls (embryonic stems) and open cotyledons (embryonic leaves). In contrast, seedlings grown in the dark have long hypocotyls and closed cotyledons forming an apical hook. This form is said to be etiolated. Genetic screens in the model plant *Arabidopsis thaliana* have made use of these distinct morphologies to identify components of light signaling pathways. One class of mutants exhibits long hypocotyls in the light and thus exhibit a decreased response to the light signal. Cloning of these positive signaling components has identified photoreceptors, which perceive the light signal, as well as downstream components such as the HY5 (ELONGATED HYPOCOTYL 5) transcription factor. A second class of mutants exhibits open cotyledons and short hypocotyls, like light-grown plants, even when grown in the dark. These mutants are called *de-etiolated (det)* or *constitutive photomorphogenic (cop)* or *fusca (fus)*, because of the high levels of the purple pigment anthocyanin. The *DET/COP/FUS* genes are negative regulators of light signaling, acting downstream of the photoreceptors but upstream of *HY5* (Chen and Chory 2011).

The founding member of the *det/cop/fus* class is *det1*. In addition to being de-etiolated in the dark, *det1* mutants exhibit defects in the light, including dwarf stature, decreased chlorophyll, increased anthocyanin, decreased apical dominance, and photoperiod independent flowering (Chory *et al.* 1989, Pepper and Chory 1997). *DET1* has been cloned and found to encode a novel nuclear protein that interacts with DAMAGED DNA BINDING PROTEIN 1A (DDB1A) and CONSTITUTIVE PHOTOMORPHOGENIC 10 (COP10) to form the CDD complex (Pepper *et al.* 1994; Schroeder *et al.* 2002; Yanagawa *et al.* 2004). The CDD complex in turn interacts with CULLIN 4 (CUL4) and RING-BOX 1 (RBX1) (Bernhardt *et al.* 2006; Chen *et al.* 2006). DET1 also interacts with histone 2B (H2B), suggesting it may be involved in regulating chromatin structure or transcription factor access (Benvenuto *et al.* 2002). Recently, DET1 was found to interact with the transcription factors CCA1 and LHY1 to act as a transcriptional repressor (Lau *et al.* 2011).

In addition to its role in visible light response, *DET1* was recently found to be involved in UV tolerance. *det1* mutants were found to be UV resistant as the result of increased levels of anthocyanin sunscreens, as well as increased expression of photolyase genes (Castells *et al.* 2010). UV light induces thymine dimers in DNA that interrupt transcription and DNA replication. These dimers can be removed via light repair, where photolyase enzymes use energy from visible light to directly cleave the dimer, or via dark or nucleotide excision repair (NER), where the lesions are recognized, unwound, removed and repaired in a multi-step process (Ganpudi and Schroeder 2011).

A key component of the NER pathway is the XERODERMA PIGMENTOSA D (XPD) helicase, which unwinds the region of UV-damaged DNA, facilitating its removal. In humans, mutation of *XPD* results in xeroderma pigmentosa, a UV-sensitive condition with increased skin cancer risk. The XPD helicase is a component of the TFIIH multi-protein complex and thus is also involved in transcription. Mutations in human *XPD* can also result in Cockayne syndrome or trichothiodystrophy, which include developmental and neurologic symptoms (Fuss and Tainer 2011).

A mutation in the Arabidopsis homolog of *XPD* was identified in a screen for UV-sensitive mutants as *UV hypersensitive 6* (*uvh6*) (Jenkins *et al.* 1995; Liu *et al.* 2003). Like human *xpd* mutants, the *uvh6-1* partial loss of function allele exhibits pleiotropic defects, including dwarf stature, decreased chlorophyll, and heat sensitivity (Jenkins *et al.* 1997). As the result of the overlapping phenotypes of *uvh6* and *det1*, we generated the double mutants to examine the interactions between these two genes with respect to light signaling, UV tolerance, and heat response.

## Materials and Methods

### Plant material and growth conditions

Throughout this study, the Arabidopsis *(Arabidopsis thaliana)* ecotype Col-0 was used as the wild-type plant. The *det1-1* partial loss of function mutant line was described previously (Chory *et al.* 1989), and the *uvh6-1* mutant line (Jenkins *et al.* 1995) (TAIR no. CS6375) was obtained from the Arabidopsis Stock Centre (http://www.arabidopsis.org/).

Unless otherwise indicated, plants were grown at 20° and 50% relative humidity. Light was supplied by cool white fluorescent bulbs with a photoperiod of 16-hr light (100 μmol photons m^−2^ s^−1^). Adult plants were grown in Sunshine mix number 1 (SunGro, Bellevue, WA).

### Growth analysis

#### Seedlings:

Seeds were plated on Linsmaier and Skoog (LS) media (Caisson) [1× LS salts, 0.8% phytoblend (Caisson), 2% sucrose], stratified at 4° for 2 days, transferred to either long-day conditions (light) or 6 hr of light then wrapped in foil (dark). After 7 days, seedlings were scanned and hypocotyl length and apical hook angle measured for dark-grown seedlings, or hypocotyl length and cotyledon width measured for light-grown seedlings, using NIH Image. Anthocyanin and chlorophyll analysis were done as previously described (Schroeder *et al.* 2002; Fankhauser and Casal 2004) using three replicates per genotype of 20 seedlings each. For gravitropism analysis, seedlings were grown in the dark on vertical plates for 7 days, then scanned and the angle between the hypocotyl and the vertical measured using NIH Image.

#### Adults:

Seeds were plated as described previously, grown in long-day conditions for 2 weeks, then transplanted to soil. Rosette diameter was measured at 4 weeks of age, and height, number of stems, and silique length were determined at 7 weeks.

### UV tolerance

Seedlings were grown on vertical plates [1× LS salts, 0.8% phytoblend (Caisson), 0.6% sucrose] in long-day conditions for 3 days, then irradiated with 600 J m^−2^ of UV-C using a Model XX-15S UV lamp (UV Products). Plates were rotated by 90°, grown in long-day for an additional 2 days, and then scanned. NIH image was used to measure new root growth beyond the bend and data expressed as relative to unirradiated controls.

### Heat tolerance

Tolerance of adult plants to heat stress was based on assays used in Jenkins *et al.* (1997). In brief, seedlings were grown on plates for 2 weeks, transferred to soil, 1week later transferred to 37° for 0–3 days, then returned to 20°. One week after the start of heat treatment, rosette diameters were measured and leaf damage scored as damaged leaves/total leaves for each plant. Tolerance of dark-grown seedlings to heat stress was determined by assays used in Larkindale *et al.* (2005). In brief, seedlings were grown in the dark on small plates with 35 mL of media [1× LS salts, 0.8% phytoblend (Caisson), 0.6% sucrose] per plate for 3 days; transferred to 45° for 0, 2, or 4 hr; then returned to 20°. After an additional 4 days of dark growth, plates were scanned and hypocotyl length measured using NIH Image.

### Heat-induced gene expression

Fifty seeds per genotype per treatment were plated on plates with 35 mL of media as described previously, stratified at 4° for 2 days, grown at 20° in long day conditions for 14 days, placed in a 45° incubator for 3 hr, allowed to recover at 20° for 1 hr, and then samples were collected. RNA was extracted using a QIAGEN RNeasy Plant Mini kit according to manufacturer instructions, including a DNase step, and quantified using a Nano-drop spectrophotometer (Thermo Scientific). cDNA was synthesized from 1 μg of total RNA using a Maxima First Strand cDNA synthesis kit (Fermentas) and diluted 40-fold for analysis. Real-time polymerase chain reaction (PCR) was performed in a 96-well plate on a iCycler equipped with iQ5 detection system (Bio-Rad) using iQ SYBR Green Supermix (Bio-Rad) in 20 μL of reaction volume. The following primers were used: At5g12030 *HSP17.6* (CCCCCTGAAGAACAAACCGAGA, TCCCTCTGTCTTTTGCCACTC), At4g27670 *HSP21* (CGCTTAACCATGGACGTCTCTC, CTGACACTCCACTTCCTCCTC), and At5g60390 *EF1α* (CTGGAGGTTTTGAGGCTGGTAT, CCAAGGGTGAAAGCAAGAAGA). For a single experiment (four genotypes ± heat treatment), samples were assayed in triplicate (technical) and values normalized relative to the reference gene *EF1α* (Jain *et al.* 2006; Hossain *et al.* 2012) then expressed as relative to the untreated wild-type control. The entire experiment was repeated three times.

### Statistical analysis

Data were compared by Student's *t*-test, and *P* values of 0.05 or less were considered to be statistically significant. All experiments were repeated at least three times.

## Results

### Dark-grown seedlings

**Figure 1:**
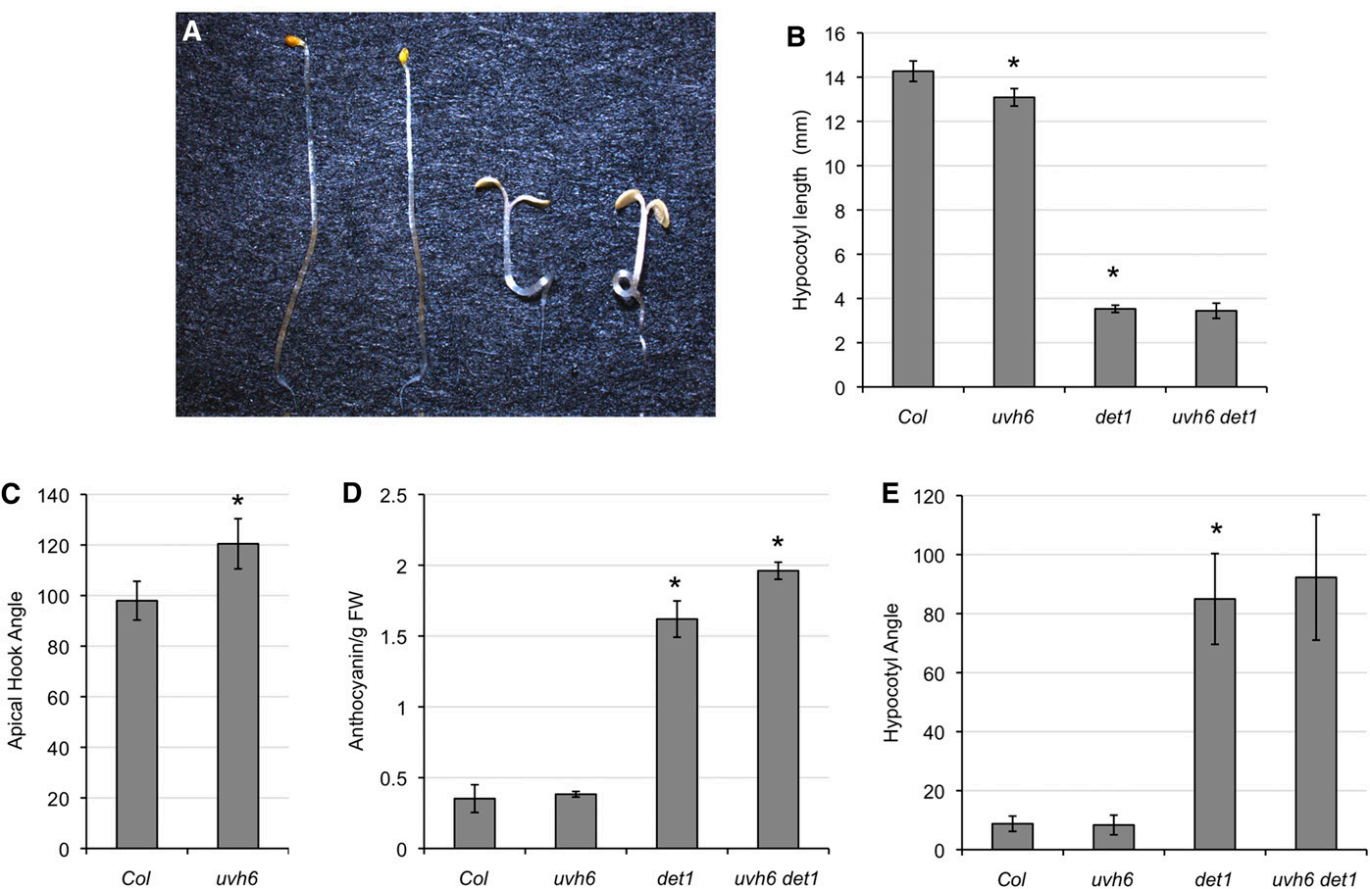
Dark-grown seedlings. (A) From left: Col-0, *uvh6*, *det1*, *uvh6 det1*. (B) Hypocotyl length (*n* = 10). (C) Apical hook angle (*n* = 10). (D) Anthocyanin content (*n* = 3). (E) Angle of hypocotyl deviation from vertical on vertical plates (*n* = 20). Error bars indicate 95% confidence interval (95% CI), and ^*^P ≤ 0.05 of single mutants relative to Col-0 or of double mutant relative to *det1*.

*det1* mutants were originally identified via their light-grown phenotype when grown in the dark (Chory *et al.* 1989). To assess the genetic relationship between *DET1* and *UVH6* with respect to de-etiolation response, *det1*, *uvh1*, and the double *det1 uvh6* mutant were grown in the dark and their phenotypes examined (Figure 1A). *uvh6* single mutants exhibited a small-but-significant decrease in hypocotyl length as well as an increase in apical hook angle (Figure 1, B and C), suggesting a weak de-etiolated phenotype. *det1* appears to be epistatic to *uvh6*, however, because the *uvh6 det1* double mutant does not differ from *det1* with regard to hypocotyl length (Figure 1B) or cotyledon opening angle (data not shown). In dark-grown seedlings, although *uvh6* does not exhibit any difference in anthocyanin levels from the wild type, it enhances anthocyanin content in the *det1* background (Figure 1D). The *det1* single and *uvh6 det1* double mutant exhibit curled hypocotyls in the dark (Figure 1A). This phenotype has previously been observed in *cop/det/fus* mutants (Hou *et al.* 1993) and indicates defects in gravitropism. Normally, in wild-type seedlings, light inhibits gravitropism (Fankhauser and Casal 2004); thus, another feature of the *cop/det/fus* phenotype is the constitutive inhibition of gravitropism in the dark. We quantified this phenotype by growing seedlings on vertical plates in the dark and measuring the angle by which hypocotyls deviated from the vertical (Figure 1E). In *det1* mutants, hypocotyl orientation was basically random. *uvh6* did not affect this phenotype in either the wild-type or *det1* background. In contrast to the shoot gravitrophic response, root gravitropism in the dark was normal in all genotypes (data not shown). In summary, in dark-grown seedlings *uvh6* single mutants exhibit slightly reduced hypocotyl length and increased apical hook opening but no change in anthocyanin content or shoot gravitropism. In the *det1* background, *uvh6* does not affect hypocotyl length or shoot gravitropism but slightly enhances anthocyanin content.

### Light-grown seedlings

**Figure 2:**
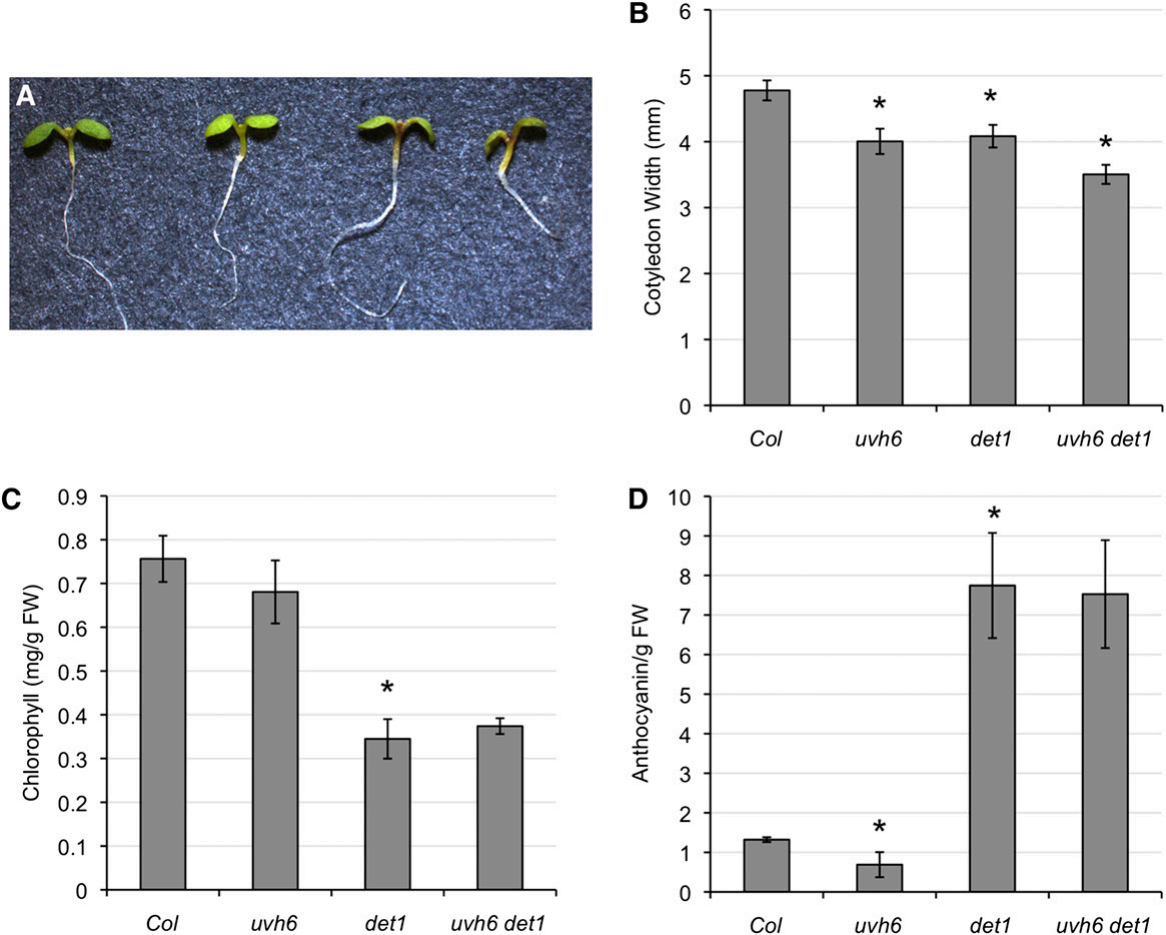
Light-grown seedlings. (A) From left: Col-0, *uvh6*, *det1*, *uvh6 det1*. (B) Cotyledon width (*n* = 20). (C) Chlorophyll content (*n* = 3). (D) Anthocyanin content (n = 3). Error bars indicate 95% CI, and ^*^P ≤ 0.05 of single mutants relative to Col-0 or of double mutant relative to *det1*.

In light-grown seedlings, *uvh6* had no detectable effect on hypocotyl length in either the wild-type or *det1* background (data not shown). However, when cotyledon width was measured, *uvh6* was found to result in decreased size in both the wild-type and *det1* backgrounds (Figure 2, A and B), indicating this effect is independent of *det1*. Both *uvh6* and *det1* mutants have been reported to be pale in color with decreased levels of chlorophyll (Chory *et al.* 1989; Jenkins *et al.* 1997). In our assay, we could not detect a significant effect of *uvh6* on chlorophyll level in either the wild-type or *det1* background (Figure 2C). Interestingly, we did detect decreased levels of anthocyanin in the *uvh6* single mutant (Figure 2D), perhaps contributing to its pale appearance. In contrast to dark-grown seedlings, anthocyanin levels did not differ between *det1* and *uvh6 det1* in light-grown seedlings.

### Adults

**Figure 3:**
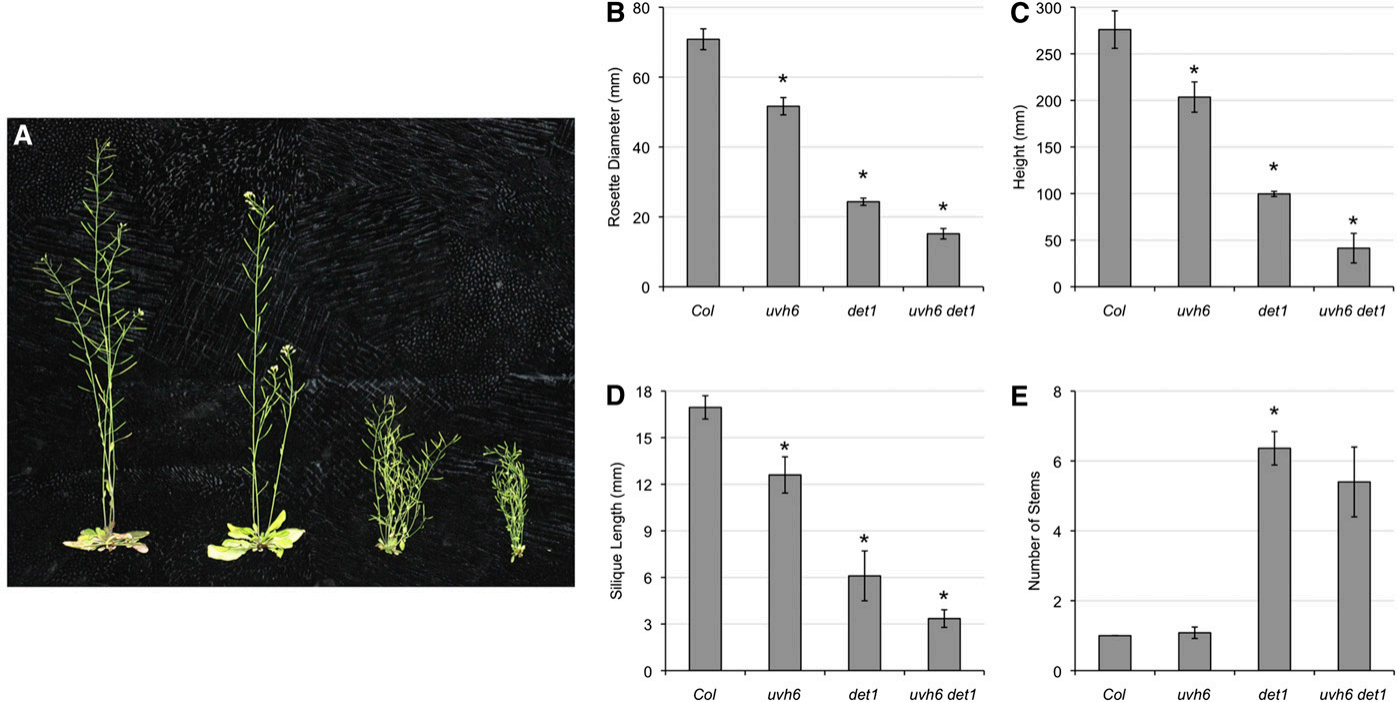
Adult phenotypes. (A) From left: Col-0, *uvh6*, *det1*, *uvh6 det1*. (B) Rosette diameter (*n* = 12). (C) Height (*n* = 12). (D) Silique length (*n* = 10). (E) Number of stems (*n* = 12). Error bars indicate 95% CI, and ^*^P ≤ 0.05 of single mutants relative to Col-0 or of double mutant relative to *det1*.

Adult plants were grown and various growth parameters examined (Figure 3A). *uvh6* did not affect flowering time as measured in either days or leaves in either the wild-type or *det1* background (data not shown). With respect to size parameters, such as rosette diameter, height, and silique length, *uvh6* resulted in decreased size in both the wild-type and *det1* backgrounds (Figure 3, A−D), indicating that the dwarf phenotypes of *uvh6* and *det1* are independent and additive. *uvh6* did not significantly affect apical dominance (Figure 3E).

### UV tolerance

**Figure 4:**
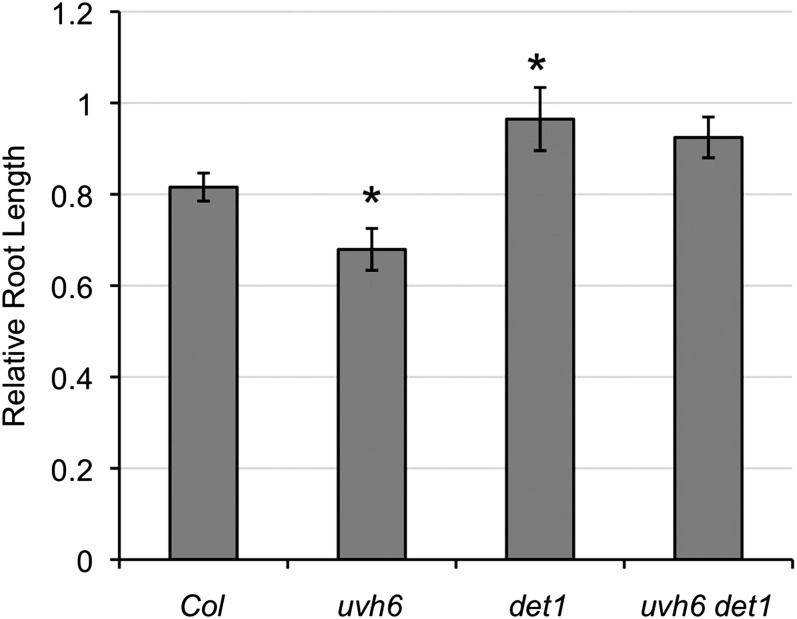
UV tolerance. Relative root length of seedlings exposed to 600 J /m^2^ UV-C then incubated in long day conditions for two days. Data are expressed as relative to unirradiated control of the same genotype (*n* = 25). Error bars indicate SE and ^*^P ≤ 0.05 of single mutants relative to Col-0 or of double mutant relative to *det1*.

The UVH6/XPD helicase is a key component of the nucleotide excision repair pathway (Fuss and Tainer 2011). The *uvh6-1* point mutant exhibits mild UV sensitivity (Jenkins *et al.* 1995) (Figure 4). *det1* mutants have recently been reported to be UV resistant as the result of photolyase overexpression (Castells *et al.* 2010). As expected, the *det1* UV-resistant phenotype is epistatic to the *uvh6*-sensitive phenotype (Figure 4) because in light conditions excess photolyase activity would compensate for defects in nucleotide excision (dark) repair.

### Heat tolerance

**Figure 5:**
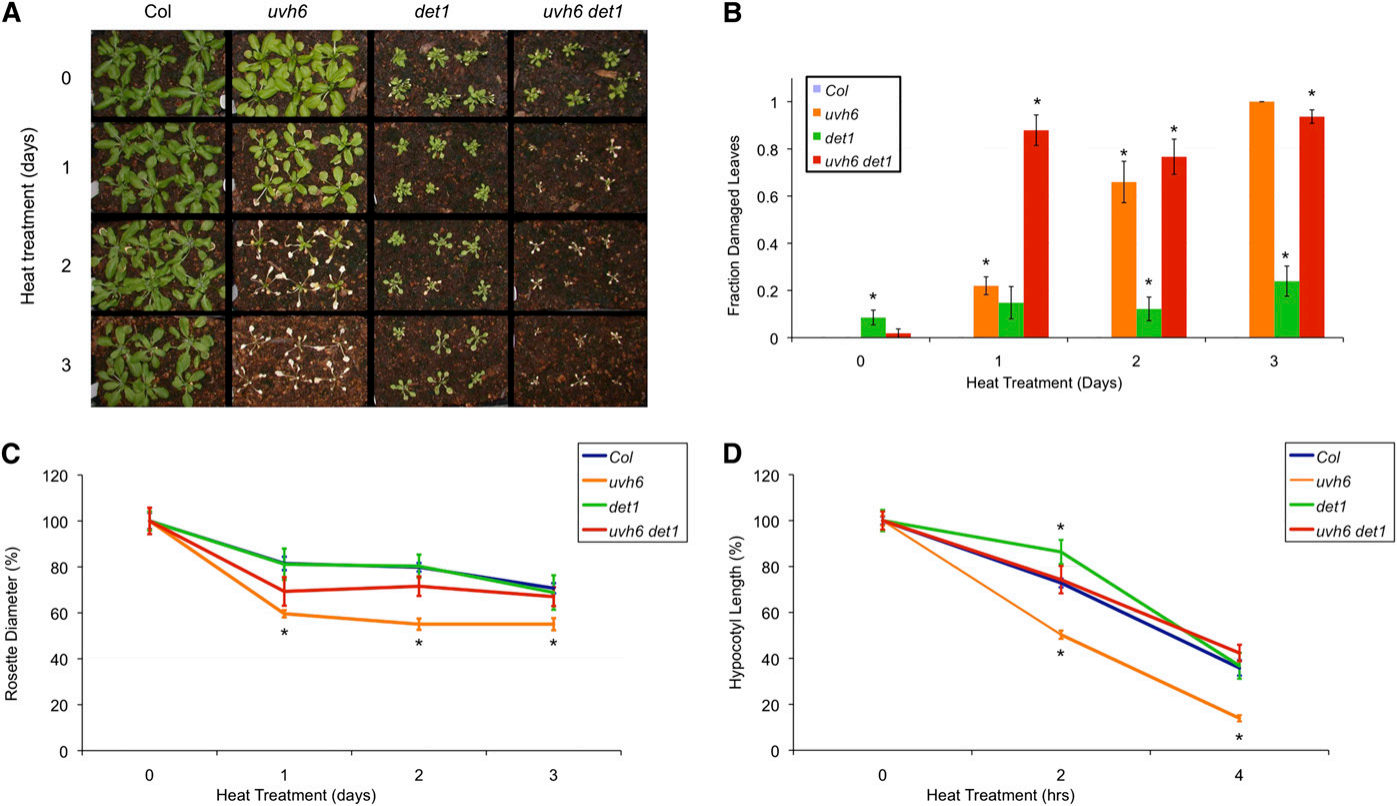
Heat tolerance. (A) Adult plants after 0−3 days of heat treatment. (B) Fraction of damaged leaves after 0−3 days of heat treatment (*n* = 6). (C) Relative rosette diameter after 0−3 days of heat treatment (*n* = 6). (D) Relative hypocotyl length after 0−4 hr of heat treatment. For C and D, data are expressed as relative to untreated controls of same genotype. For B−D, error bars indicate SE, and ^*^P ≤ 0.05 of single mutants relative to Col-0 or of double mutant relative to *det1* in the same conditions.

*uvh6* mutants show increased heat sensitivity (Jenkins *et al.* 1997). We examined heat tolerance in *uvh6*, *det1*, and the double mutant. In adult plants, 2 and 3 days of heat treatment killed the *uvh6* single mutant but did not result in significant leaf damage in wild type (Figure 5, A and B). *det1* mutants exhibited low levels of leaf damage even in control conditions; however, this was not significantly increased by heat treatment. In the *uvh6 det1* double mutant, 1 day of heat treatment resulted in dead plants, indicating that *det1* enhanced heat-induced tissue damage in *uvh6*. Another effect of heat is inhibition of growth. To quantify this effect, we measured rosette diameters in all genotypes and treatments and calculated relative rosette diameter (Figure 5C). Heat treatment resulted in a significant reduction in rosette diameter in *uvh6* relative to the wild type. In *det1*, heat inhibition of growth was similar to that observed in wild type. In *uvh6 det1*, relative rosette diameter was intermediate between the two single mutants. It was not significantly different from *det1* in any condition and was significantly greater than that of *uvh6* after 2 and 3 days of heat treatment. Thus, in adult plants, *det1* suppressed heat inhibition of growth in *uvh6* while enhancing heat-induced tissue damage. To investigate heat tolerance at other stages of development, we examined the effect of heat on hypocotyl length in dark-grown seedlings (Figure 5D). Again, *uvh6* exhibited increased growth inhibition relative to the wild type. *det1* mutants exhibited slightly decreased inhibition relative to the wild type at intermediate treatment duration. As in the adult assay, the *uvh6 det1* double mutants were not significantly different from *det1* in any condition but exhibited significantly less inhibition than *uvh6* in both heat treatments. Therefore, in both adults and dark-grown seedlings, *det1* suppressed heat inhibition of growth in *uvh6*.

### Heat regulation of gene expression

**Figure 6:**
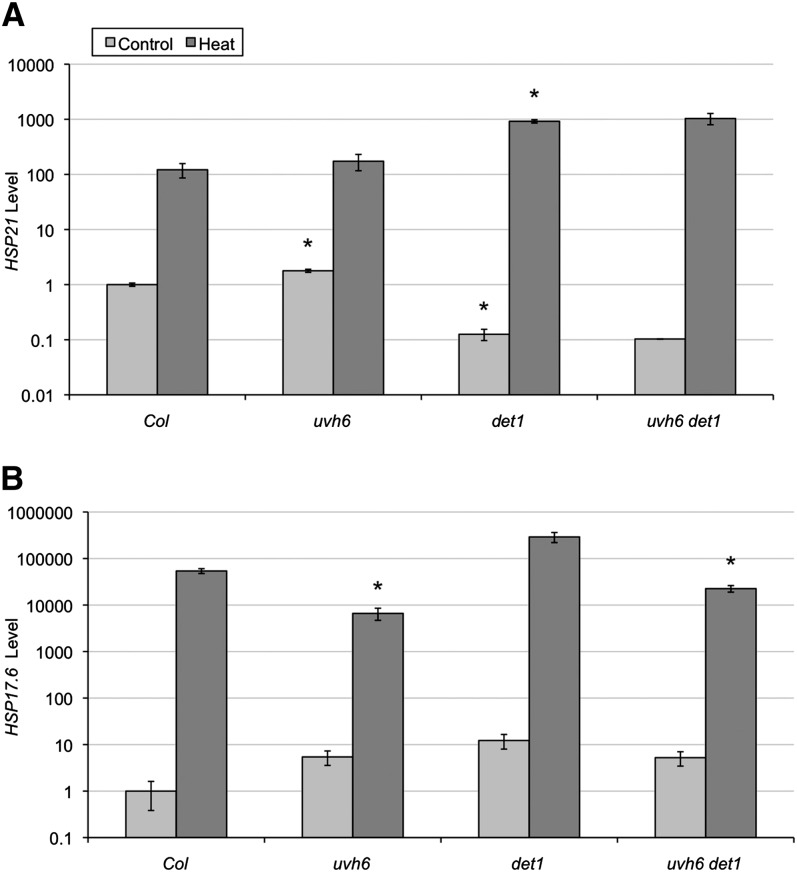
Heat-induced gene expression. mRNA levels of *HSP21* (At4g27670) (A) and *HSP17.6* (At5g12030) (B) in the absence and presence of heat treatment (3 hr 45° + 1 hr 20°) as determined by real-time reverse-transcription PCR. Values were normalized relative to the reference gene *EF1α* then expressed as relative to the untreated wild-type control. Error bars indicate SE (*n* = 3) and ^*^P ≤ 0.05 of single mutants relative to Col-0 or of double mutant relative to *det1* in the same conditions.

As a component of TFIIH, XPD/UVH6 plays an important role in transcription. *uvh6* mutants have been reported to exhibit aberrant levels of several RNAs and proteins (Jenkins *et al.* 1997; Liu *et al.* 2008; Hall *et al.* 2009). With respect to its role in heat tolerance, *uvh6* was reported to contain increased levels of HSP21 (Jenkins *et al.* 1997) but normal levels of HSP101 and sHSPs (Larkindale *et al.* 2005). *det1* mutants also misexpress hundreds of genes. Interestingly, many heat shock protein genes are overexpressed in *det1* mutants in light conditions (supporting information, Table S1) (Maxwell 2001; Schroeder *et al.* 2002; Ma *et al.* 2003). We examined expression levels of several heat shock protein genes in light-grown seedlings with or without heat treatment using real-time reverse-transcription PCR. HSP21 protein was previously found to be present at increased levels in *uvh6* mutants (Jenkins *et al.* 1997). An increase in At4g27670 *HSP21* transcript levels was detected in untreated *uvh6* seedlings (Figure 6A). In contrast, *HSP21* levels were lower in untreated *det1* and *uvh6 det1* than in the wild type. After heat treatment, however, *HSP21* levels were greater in *det1. uvh6* did not appear to affect *HSP21* levels after heat treatment in either the wild type or *det1* background. At5g12030 *HSP17.6* encodes a class 1 small HSP, which had previously been shown to be unchanged in *uvh6* mutants (Larkindale *et al.* 2005). We observe a decrease in induced *HSP17.6* levels in both *uvh6* relative to wild type and in the double mutant relative to *det1* (Figure 6B).

## Discussion

In this study we examined the genetic interactions between the pleiotropic *det1* and *uvh6* mutations. In dark-grown seedlings, *uvh6* exhibited a mild de-etiolated phenotype, consisting of a slight decrease in hypocotyl length and an increase in apical hook angle. *det1* appears to be epistatic to this phenotype because the *uvh6 det1* double mutants do not differ from *det1* with respect to hypocotyl length or agravitropism. The double mutants do, however, exhibit an increase in anthocyanin in the dark relative to *det1*, indicating a mild enhancement of this phenotype. In contrast, in light-grown seedlings, *uvh6* single mutants exhibit decreased anthocyanin levels relative to the wild type, perhaps contributing to their pale appearance. *det1* is epistatic to this phenotype because *uvh6* does not affect *det1* anthocyanin levels in the light. The basis of this differential effect of light on *uvh6* anthocyanin regulation is unknown.

*det1* is best known for overexpressing light-regulated genes in the dark (Chory *et al.* 1989), but in the light it actually underexpresses light-regulated genes such as *CAB1*, *CAB2*, and *LHCB2.4* (Chory and Peto 1990; Schroeder *et al.* 2002). *DET1* regulation of the *CAB2* promoter in the light requires a HY5-binding element (Maxwell *et al.* 2003), and *hy5* mutants suppress the *det1* pale phenotype (Chory 1992). *uvh6* has been described as yellow−green with decreased chlorophyll level and poorly organized thylakoid membranes (Jenkins *et al.* 1997; Liu *et al.* 2003). In seedlings, however, we did not detect a significant effect of *uvh6* on chlorophyll levels in either the wild-type or *det1* background.

For all parameters that describe plant size in light conditions, such as light-grown seedling cotyledon width, as well as adult rosette diameter, height, and silique length, *uvh6* and *det1* act additively. Although the basis of the *det1* dwarf phenotype is not entirely clear, it is partially suppressed by the *ted* mutants, including *hy5 (ted5)* and the peroxisomal protein gene *ted3*, suggesting that transcription and peroxisome function play a role (Chory 1992; Pepper and Chory 1997; Hu *et al.* 2002). The basis of the *uvh6* dwarf phenotype is also unknown, but nonetheless these data suggest that *UVH6* acts independently of *DET1* to regulate plant size.

*uvh6* mutants exhibit strong heat sensitivity; however, the role of *UVH6* in heat tolerance is distinct from known heat tolerance pathways (Jenkins *et al.* 1997; Larkindale *et al.* 2005; Kotak *et al.* 2007). Here we show that *det1* enhances heat-induced tissue damage in *uvh6*. In many ways light-grown *det1* behave like light stressed plants, with decreased chlorophyll, increased anthocyanin, and photosynthetic rates and chlorophyll composition typical of plants grown in high light (Walters *et al.* 1999), as well as increased levels of genes associated with light stress such as photolyases (Hu *et al.* 2002; Castells *et al.* 2010). Light stress can induce cell death in plants, and the blue light receptor CRY1 is required for this response (Danon *et al.* 2006). *det1* exhibits constitutive light signaling in a number of pathways, including CRY1. Combined heat and light treatments result in reduced plant survival (Larkindale and Knight 2002; Larkindale *et al.* 2005). Thus, perhaps the decreased survival in the *det1 uvh6* double mutants is attributable to the combination of *det1* constitutive light stress response with *uvh6* heat sensitivity. In contrast to the enhanced heat-induced tissue damage in the *uvh6 det1* double mutants, *det1* suppresses heat-induced growth inhibition in the *uvh6* background. These differential effects may be attributable to the nature of the *det1* phenotype. *det1* mutants are small, stressed plants. When combined with the heat sensitivity of the *uvh6* mutants, the double mutants are hypersensitive to heat stress at the cellular level but do not exhibit additional heat-induced growth inhibition. These data suggest that basis for being small in *det1* is epistatic to heat-induced inhibition of growth in *uvh6*.

With respect to heat regulation of gene expression, for *HSP21* we detect increased levels in *uvh6* mutants, consistent with previous studies showing increased HSP21 proteins levels (Jenkins *et al.* 1997). For *HSP17.6*, however, we detect reduced levels in *uvh6* mutants, in contrast to the unchanged amounts of class 1 sHSP protein previously described (Larkindale *et al.* 2005). This difference could be the result of differential regulation of RNA *vs.* protein or differences in developmental stage or heat treatment. Although in some studies authors indicate enhanced response to heat treatment by *uvh6* (*e.g.*, Jenkins *et al.* (1997)), others observe reduced effects. For example, Liu *et al.* (2008) show that heat treatment reduced levels of *AtKu70* and *AtKu80* transcript in wild type, but this down-regulation did not occur in *uvh6*. In response to another stress, cold, Hall *et al.* (2009) found that *uvh6* mutants failed to induce some cold stress genes but not all. Thus the *uvh6-1* mutant appears to exhibit abnormal regulation of a subset of genes rather than global defects in transcription (Hall *et al.* 2009). In *det1* mutants, we also detect abnormal levels of HSP transcripts, consistent with previous studies implicating DET1 in regulation of gene expression (Benvenuto *et al.* 2002; Schroeder *et al.* 2002; Ma *et al.* 2003; Lau *et al.* 2011). Whether this phenotype is attributable to variation in transcription factor abundance, chromatin structure, or direct regulation of transcription is still unclear. Nonetheless, neither of the genes examined exhibit enhanced levels in the double mutant, suggesting that the enhanced heat induced tissue damage in the double mutant is not due to a global increase in heat response.

In summary, we find that *det1* acts epistatically to *uvh6* with respect to de-etiolation, chlorophyll level, UV tolerance, and heat-induced growth inhibition. Interestingly, many of these *det1* phenotypes have been shown to require HY5 activity (Chory 1992; Pepper and Chory 1997; Maxwell *et al.* 2003; Castells *et al.* 2010). Perhaps transcriptional regulation via HY5 is the basis of *det1* epistasis. In contrast, *det1* and *uvh6* act additively to regulate plant size and heat-induced tissue damage, suggesting that these traits are regulated by independent (probably indirect transcriptional) means. Thus *DET1* and *UVH6* act in both common and independent pathways to regulate plant response to light and heat.

## Supplementary Material

Supporting Information
