# Supplementary material for: Genetic Interactions Between Arabidopsis DET1 and UVH6 During Development and Abiotic Stress Response
Source: G3 (Bethesda). 2012 Aug 1;2(8):913–20. doi: 10.1534/g3.112.003368 (PMC3411247; doi:10.1534/g3.112.003368)
Supplement: Supporting Information [file supp_2_8_913__index.html]

Supporting Information 

# Genetic Interactions Between Arabidopsis *DET1* and *UVH6* During Development and Abiotic Stress Response

## Supporting Information for Kim *et al.*, 2012

**Files in this Data Supplement:**

- Table S1 - Heat-related genes with more than a three-fold difference in expression level in det1 relative to wildtype Col-0 in dark or after 3h light treatment (PDF, 63 KB)
