# Supplementary material for: Genetic Interactions Between Arabidopsis DET1 and UVH6 During Development and Abiotic Stress Response
Source: G3 (Bethesda). 2012 Aug 1;2(8):913–20. doi: 10.1534/g3.112.003368 (PMC3411247; doi:10.1534/g3.112.003368)
Supplement: Supporting Information [file supp_2.8.913_003368SI.pdf]

Table S1 Heat-related genes with more than a three-fold difference in expression level in *det1* relative to wildtype Col-0 in dark or after 3h light treatment. Compiled from Maxwell (2001) and Schroeder *et al.* (2002).

**Genes Overexpressed in Dark-Grown *det1***	
At1g23100	putative 10kd chaperonin -06.04 protein targeting, sorting and translocation HSP10 - chaperonin, mitochondrial AFFY Arabidopsis thaliana chromosome I BAC T26J12 genomic sequence, complete sequence.
At1g53540	17.6 kDa heat shock protein (AA 1-156) -11.01 stress response heat shock protein 22.0 AFFY Arabidopsis thaliana hsp17.6 mRNA for 17.6 kDa heat shock protein.
At2g04030	putative heat shock protein -30.03 organization of cytoplasm HSC82 - heat shock protein AFFY Arabidopsis thaliana chromosome II BAC F3C11 genomic sequence, complete sequence.
**Genes Underexpressed in Dark-Grown *det1***	
At2g27140	unknown protein -11.01 stress response heat shock protein 22.0 AFFY Arabidopsis thaliana chromosome II BAC T20P8 genomic sequence, complete sequence.
At4g36990	heat shock transcription factor HSF4 JP7_9D09L.41.65.x Exon
**Genes Overexpressed in Light-Treated *det1***	
At1g07400	putative heat shock hsp20 protein.
At1g53540	17.6 kDa heat shock protein (AA 1-156) -11.01 stress response heat shock protein 22.0 AFFY Arabidopsis thaliana hsp17.6 mRNA for 17.6 kDa heat shock protein.
At1g54050	hypothetical protein -11.01 stress response heat shock protein 22.0 AFFY Sequence of BAC F15I1 from Arabidopsis thaliana chromosome 1, complete sequence.
At1g61360	heat shock transcription factor 21.
At1g74310	heat shock protein 101 -11.01 stress response HSP104 - heat shock protein AFFY Arabidopsis thaliana heat shock protein AtHSP101 (Athsp101) mRNA, complete cds.
At2g29500	putative small heat shock protein -11.01 stress response heat shock protein 22.0 AFFY Arabidopsis thaliana chromosome II BAC F16P2 genomic sequence, complete sequence.
At2g33210	mitochondrial chaperonin (HSP60) -06.01 protein folding and stabilization HSP60 - heat shock protein - chaperone, mitochondrial AFFY Arabidopsis thaliana chromosome II BAC F25I18 genomic sequence, complete sequence.
At3g46230	heat shock protein 17 - AFFY Arabidopsis HSP17.4 gene for 17.4kDa heat shock protein.
At4g10250	heat shock protein 22.0 -11.01 stress response heat shock protein 22.0 AFFY Arabidopsis thaliana Columbia endomembrane-localized small heat shock protein AtHSP22.0 mRNA, complete cds.
At4g18880	heat shock transcription factor - like protein -11.01 stress response heat shock transcription factor - like protein AFFY Arabidopsis thaliana heat shock transcription factor 21 (AtHSF21) mRNA, partial cds.
At4g24190	HSP90-like protein -11.01 stress response HSP90-like protein AFFY Arabidopsis thaliana chromosome IV BAC T19F6 genomic sequence, complete sequence.
At4g25200	AtHSP23.6-mito.
At4g35770	senescence-associated protein sen1 -06.04 protein targeting, sorting and translocation similarity to D.melanogaster heat shock protein 67B2 AFFY Arabidopsis thaliana senescence-associated protein mRNA, complete cds.
At4g37910	heat shock protein 70 like protein -11.01 stress response heat shock protein 70 like protein AFFY Arabidopsis thaliana DNA chromosome 4, BAC clone F20D10 (ESSA project).
At5g12020	heat shock protein 17.6-II -11.01 stress response heat shock protein 22.0 AFFY A.thaliana mRNA for heat shock protein hsp 17.6 - II.
At5g12030	heat shock protein 17.6A -11.01 stress response heat shock protein 21 AFFY Arabidopsis thaliana mRNA for heat shock protein 17.6A.
At5g42020	luminal binding protein (dbj|BAA13948.1) -06.01 protein folding and stabilization SSA2 - heat shock protein of HSP70 family, cytosolic AFFY Arabidopsis thaliana mRNA for luminal binding protein (BiP), complete cds.
At5g52640	heat-shock protein -11.01 stress response HSP90-like protein AFFY A.thaliana heat shock protein 83 mRNA, complete cds.
